# Predictors of pre-operative cognitive impairment in meningioma patients over 60 years old

**DOI:** 10.1186/s12883-020-01806-0

**Published:** 2020-06-03

**Authors:** Min Ju Kang, Jung-Min Pyun, Min Jae Baek, Kihwan Hwang, Jung Ho Han, Young Ho Park, Chae-Yong Kim, SangYun Kim

**Affiliations:** 1 Department of Neurology, Veterans Medical Research Institute, Veterans Health Service Medical Center, Seoul, Republic of Korea; 2 Department of Neurology, Seoul National University Bundang Hospital, 82, Gumi-ro 173 Beon-gil, Bundang-gu, Seongnam-si, Gyeonggi-do 13620 Republic of Korea; 3 Department of Neurology, Seoul National University College of Medicine, Seoul, Republic of Korea; 4 Department of Neurosurgery, Seoul National University Bundang Hospital, 82, Gumi-ro 173 Beon-gil, Bundang-gu, Seongnam-si, Gyeonggi-do 13620 Republic of Korea; 5 Department of Neurosurgery, Seoul National University College of Medicine, Seoul, Republic of Korea

**Keywords:** Cognitive dysfunction, Meningioma, Neuropsychological tests

## Abstract

**Background:**

The aim of this study was to assess the cognitive function of patients over 60 years old with meningioma using a domain-specific neuropsychological test and to investigate the relevant factors affecting pre-operative cognitive decline in different subdomains.

**Methods:**

We retrospectively investigated 46 intracranial meningioma patients between the ages of 60 and 85 years. All patients underwent brain MRI and neuropsychological test. Tumor size, location, peritumoral edema, and medial temporal atrophy (MTA) were examined to determine the association with cognitive impairment. We performed a logistic regression analysis to examine the odds ratios (ORs) for cognitive decline of four subdomains: verbal memory, language, visuospatial, and executive functions.

**Results:**

Tumor size and age were associated with executive dysfunction (OR 1.083, 95% confidence interval (CI) 1.006–1.166, and OR 1.209, 95% CI 1.018–1.436, respectively). There was no statistically significant association in other cognitive domains (language, verbal memory, and visuospatial function) with variables in regression analysis.

**Conclusions:**

We conclude that tumor size and age are positively related with executive function in pre-operative meningioma patients over 60 years old.

## Background

Meningioma is a frequently observed brain tumor in elderly patients [1]. It has been shown to be associated with alterations in cognitive function, often masquerading dementia [2]. To strategize the best treatment approach, a prediction of cognitive function with relevant factors is crucial. However, cognitive deficits are largely variable and cannot be explained by the localization of tumor alone [3]. There have been previous efforts to investigate the relevance of tumor variables, but the results were inconsistent [4].

A significant portion of elderly patients may undergo neurodegeneration, which is the process of neuronal injury [5]. Alzheimer’s disease is the most common neurodegenerative disease that causes cognitive alteration [6], and medial temporal atrophy (MTA) observed on structural magnetic resonance imaging (MRI) is widely used as a biomarker [7]. Not only the tumor variables, but the underlying conditions, such as neurodegeneration, may influence the extent of cognitive functioning that may lead to the inter-individual variation of cognitive impairments.

In this study, we retrospectively examined the pre-operative cognitive function of patients with meningioma over 60 years old using domain-specific neuropsychological test and investigated the tumor variables, including tumor location, size, and peritumoral edema, affecting cognitive decline in various subdomains. Moreover, we also examined the severity of MTA, which is the biomarker of Alzheimer’s disease, and whether they are a relevant factor influencing cognitive function in meningioma.

## Methods

Between December 2013 and November 2017, patients previously diagnosed with intracranial meningioma were recruited from Seoul National University Bundang Hospital. Patients aged between 60 and 85 years and with history of neuropsychological tests were included. Those without brain MRI and with history of intracranial surgery or radiotherapy prior to receiving neuropsychological tests were excluded.

### Cognitive assessments

Demographic data and clinical information were obtained from the retrospective medical record reviews. The Mini-Mental State Examination (MMSE) was used to evaluate global cognitive impairment. We obtained extensive neuropsychological assessments of various domains, including tests for language, verbal memory, visuospatial, and executive function. We assessed each cognitive domain using the following tests: the Korean short version of Boston Naming Test [8] for language; the Rey Complex Figure Test [9]- copy for visuospatial function; the Seoul Verbal Learning Test [10]-delayed recall for verbal memory; and color reading which was number of correctly answered color of the ink (instead of reading the word) within 60 s of Stroop Test [11] for executive function. In this study, scores below 1 standard deviation of the age, gender, and education specific norm were classified as abnormal.

### MRI

Tumor size and peritumoral edema were determined by MRI. The time interval between brain MRI and neuropsychological tests was restricted to less than 1 month. The median time interval between the two tests was 4 days (Interquartile range 2–17 days). Tumor size was identified as the largest diameter which was measured from contrast-enhanced tumor on the postgadolinium T1-weighted axial and/or coronal image of the tumor. The extent of peritumoral edema was identified as high signal intensity on a T2-weighted MRI; the extent of peritumoral edema was graded from 0 to 2, where grade 0 represented either absence of edema or the presence of a small halo around the tumor; grade 1 represented edema extending variably along the tracts of the white matter but without involvement of the whole hemisphere; and grade 2 represented holohemispheric or near holohemispheric edema [12]. MTA was visually assessed using a standardized scale, previously developed by Scheltens et al. [7]. Atrophy was rated on a 5-point scale (0 = absent, 1 = minimal, 2 = mild, 3 = moderate, 4 = severe) – based on the height of hippocampal formation, width of the choroidal fissure, and temporal horn at the coronal plane image. All MRI images were assessed independently by three experienced neurologists, who were blinded to clinical information. Three raters measured the tumor size, peritumoral edema, and atrophy scales for the left and right hemispheres, respectively. For the analysis of MTA, higher atrophy score between the left and right hemisphere were used. If MTA could not be analyzed due to the extent to tumor size, score from one hemisphere was used. MTA score was dichotomized into normal (0–1) and abnormal (2 or higher), and peritumoral edema grade was dichotomized into mild (0–1) and severe [2] peritumoral edema for further analysis. In the case of discrepancy between the raters, the final score was determined via consensus among the raters. Intra-rater reliability was assessed by the re-rating of MRI scans of all participants at a separate sitting, blinded to prior rating. Inter-, and intra-rater reliabilities were measured by calculating the interclass correlation coefficient.

### Statistical analysis

The comparison of baseline characteristics between study subjects and excluded ones was performed using the Student t-test for continuous variables and Pearson’s chi-square test for categorical variables. To determine inter and intra-rater reliability of visual rating scale, all the raters measured the tumor size, peritumoral edema, and MTA.

To examine the association of cognitive impairment, univariate logistic regression analysis was performed in four domains: verbal memory, language, visuospatial, and executive domains. For further analysis, multivariate logistic regression analysis was conducted to determine the independent predictors associated with cognitive decline of various domains. We performed multivariate logistic regressions with backward stepwise analysis using an entry probability of 0.05 and a removal probability of 0.10. As the hypothesis was tested separately in four independent domains, the alpha level of 0.0125 was applied after Bonferroni correction. Variance inflation factors (VIFs) were calculated among the included variables to check for multicollinearity. All statistical analyses were performed using SPSS version 22 (SPSS Inc., Chicago, Illinois). The study protocol was approved by the Institutional Review Board of Seoul National University Bundang Hospital (B-1809/493–112).

## Results

A total of 51 patients were identified as having intracranial meningioma and met the inclusion criteria. Two patients were excluded because they did not have brain MRI scans, and three patients excluded due to history of intracranial surgery or radiotherapy prior to receiving the neuropsychological test. Thus, our study population consisted of 46 patients. Subject demographics and tumor characteristics are summarized in Table 1. The mean age was 69 years, and there was female prominence (60%). Ten patients (21.7%) showed abnormal score of MTA, and 16 patients (34.8%) showed severe peritumoral edema. In a significant portion of patients (26%, 12 patients), MTA was measured unilaterally due to the extent of tumors. The interrater reliability study showed good agreement between raters for MTA (Fleiss kappa values of 0.731), peritumoral edema (Fleiss kappa values of 0.724) and tumor size (Intraclass Correlation Coefficient of 0.935). The intrarater reliability values also showed good reliability among all three raters (Cohen’s kappa values of 0.763, 0.934 and 0.827 for raters 1, 2, and 3, respectively, for MTA; weighted Kappa value of 0.723, 0.95 and 0.73 for raters 1, 2 and 3, respectively, for peritumoral edema; Intraclass Correlation Coefficient value of 0.968, 0.995 and 0.969 foe rater 1, 2 and 3, respectively, for tumor size). The VIFs were less than 1.719 for all variables, indicating a low degree of collinearity.

**Table 1:** Demographics and clinical characteristics

Female	30 (65.22)
Age in years, mean (SD)	69.59 (5.89)
Years of education, median (IQR)	12.00 (8–16)
Tumor size in mm, mean (SD)	45.12 (14.58)
Peritumoral edema	
0	9 (19.56)
1	21 (45.65)
2	16 (34.78)
MTA	
0	26 (56.52)
1	10 (21.74)
2	8 (17.39)
3	2 (4.35)
Supratentorial/infratentorial	45/1
Site of origin of meningiomas	
Skull base	12 (26.09)
Convexity	26 (56.52)
Falx	2 (4.35)
Convexity/falx	3 (6.52)
Intraventricular	3 (6.52)
Location of meningiomas^a^	
Frontal	22 (34.78)
Temporal	6 (13.04)
Parietal	4 (8.70)
Occipital	2 (2.17)
Lateralization	
Left	24 (52.17)
Right	20 (43.48)
Bilateral	2 (4.35)
Impaired cognitive domain	
Verbal memory	27 (58.70)
Language	28 (60.87)
Visuospatial function	31 (67.39)
Executive function	28 (60.87)

Data are presented as number (%) unless otherwise specified

Impaired cognitive domain was defined as scores below 1 standard deviation of the age, gender, and education specific norm

*IQR* interquartile range, *MTA* medial temporal atrophy, *SD* standard deviation

^a^Data for 12 cases were excluded since the tumor was not extended to cerebrum (3 intraventricular, 8 skull base, 1 infratentorial)

In the univariate logistic regression analysis, the age and tumor size showed significantly deteriorated performance in executive function with an odds ratio [OR] of 1.153, 95% confidence interval [CI] of 1.014–1.312, and OR (95% CI) of 1.090 (1.022–1.162) (Table 2). Multivariate logistic regression in backward stepwise analysis included clinical variables (age, sex, education), tumor size, tumor localization, MTA, and peritumoral edema. The adjusted covariates did not alter the significance of tumor size (OR 1.083; 95% CI 1.006–1.166; *p* = 0.01) and age (OR 1.209; 95% CI 1.018–1.436; p = 0.01) in association with executive dysfunction (Table 2). In contrast, there was no statistically significant association in other cognitive domains (language, verbal memory, and visuospatial function) with variables in regression analysis.

**Table 2:** Odds ratios (95% CI) for associations with executive dysfunction in univariate and multivariate logistic regression analysis

Variables	Univariate model	Multivariate model^a^
Men	0.442 (0.084–2.950)	Removed
Age	1.123 (0.962–1.310)	1.209 (1.018–1.436)
Education	1.176 (0.870–1.591)	Removed
MTA		
Normal (0–1)	Reference	
Abnormal (> 2)	2.889 (0.242–34.488)	Removed
Size	1.065 (0.998–1.135)	1.083 (1.006–1.166)
Peritumoral edema		
Mild (0–1)	Reference	
Severe (2)	2.171 (0.242–34.488)	Removed
Localization-Location		
Non-convexity	Reference	
Convexity	1.754 (0.302–10.072)	Removed
Localization-lobe^b^		
Non-frontal	Reference	Removed
Frontal	1.437 (0.171–12.046)	
Localization-lateralization^c^		
Right	Reference	Removed
Left	0.367 (0.43–3.159)	

*CI* confidence interval, *MTA* medial temporal atrophy, *SD* standard deviation

^a^The odds ratios were obtained by multivariate logistic regression with backward stepwise analysis. Gender, MTA, year of education, peritumoral edema, localization were entered into the model but were removed through the stepwise regression process

^b^Data for 12 cases were excluded since the tumor was not extended to cerebrum (3 intraventricular, 8 skull base, 1 infratentorial)

^c^Data for 2 were excluded since the tumor was located bilaterally

## Discussion

The present study demonstrated that the executive function was positively correlated with age and tumor size. Tumor size reflects the degree of injury in nerve tissues due to mechanical damage and secondary ischemia. In high-grade tumors, it is well known that the severity of neuropsychological deficits is positively associated with lesion size [13]. However, tumor histology itself does not influence the type of cognitive impairment [14], and even less invasive, low-grade tumors has also been shown to disrupt the neural network [15]. Executive function is integrated into frontal cortical and subcortical connection (fronto-subcortical systems) [16], and lesions located in this connection may result in the executive function deficits. With tumor growth, there is greater pressure on the adjacent nerve tissue, resulting in executive dysfunction. According to the present study, this association may exist with meningioma. Previous study about meningioma also demonstrated that patients with a large tumor (bigger than 4 cm in maximum diameter) had poorer performance with respect to language, fine motor, controlled oral word association test, and trail making test part B performance [17]. Meningioma growth, although slow, may be associated with cognitive impairment.

This study also demonstrated that age was positively correlated with executive dysfunction. Cognitive reserve –individual’s resistance to cognitive impairment that arises as a consequence of brain pathology – decreases as part of the normal aging process [18]. Similarly, Krupp et al. reported a rapid decline of all measured cognitive parameters in post-operative meningioma patients after the age of 55 years [19].

Moreover, the pathologic process of neurodegeneration also has a significant effect on cognitive reserve or plasticity [20]. MTA is a result of nerve cell loss and gliosis in the hippocampus, which is the most severely affected structure in Alzheimer’s disease in early stage. A significant portion (21.7%) of patients demonstrated MTA; however, a logistic regression analysis showed no relationship between MTA with cognitive function, suggesting that MTA alone might not be an important risk factor for cognitive dysfunction.

Peritumoral edema is detected in 40–75% of meningioma patients, and its presence is related to severe neurologic deficit [21]. Cerebral blood flow and cerebral blood volume are also significantly low in the peritumoral edema [22]. Previous study has reported that the apathy is much severe in patients with brain edema of medial frontal meningioma [23], and preoperative cerebral edema in WHO grade I meningioma patients is correlated with post operative cognitive dysfunction [24]. In contrast, Tucha et al. showed preoperative edema was not associated with cognitive function after surgery [25]. Both tumor and peritumoral edema exert mechanical effects on the cerebrum, and the combination may have negative effects on cognitive outcome. In this study, logistic regression analysis showed no relationship between peritumoral edema and cognitive function. Tumor size may have a stronger association with poor executive cognitive function when compared with preoperative peritumoral edema.

In this study, we did not find any differences in cognitive functions by location of the meningioma. Specifically, no cognitive function relationships were identified between the frontal and non-frontal meningiomas, as well as between convexity, non-convexity, and lateralization. Although the study design and classification of location varied, number of previous studies reported no differences in preoperative cognitive functioning based on meningioma locations [26–29], and lateralization [25–28]. In contrast, Tucha et al. showed different post-operative cognitive improvement outcomes between localization groups for various cognitive domains [25]. Meskal et al. reported better performance for complex attention in the infratentorial group than the supratentorial group in postoperative states [26], and Koizumi and Dijkstra reported that patients with right-sided meningioma showed more favorable postoperative outcomes than left-sided meningioma [27, 30]. Despite these mixed findings, meningioma location may affect post-operative cognitive outcomes. The impact of meningioma location on cognitive function should not be overlooked, and further investigation may be needed.

This study has potential limitations. First, this is considered to be a preliminary study due to the small sample size. Future study with greater number of participants is required. Second, only the presence MTA was measured as a marker of neurodegeneration. Additional study using other markers, such as 18-Fluoro-deoxyglucose positron emission tomography or amyloid positron emission tomography, may be needed.

## Conclusions

We conclude that tumor size and age are positively related with executive dysfunction in pre-operative meningioma patients over 60 years old. Based on our findings, clinicians may consider testing the executive function in these patients to better detect cognitive dysfunction.

## Data Availability

The datasets used and/or analysed during the current study are available from the corresponding author on reasonable request.

## Abbreviations

CI: Confidential intervals
MTA: Medial temporal atrophy
MMSE: Mini-mental status examination
ORs: Odds ratios
VIFs: Variance inflation factors
